# The value of thrombus markers applied in patients with respiratory failure

**DOI:** 10.5937/jomb0-50697

**Published:** 2025-01-24

**Authors:** Chen Yingqun, Yin Zihan, Wang Junshi, Yan Cunliang, Lin Xuwei, Huang Lei

**Affiliations:** 1 Peking University Shenzhen Hospital, Department of Critical Care Medicine, Shenzhen, China; 2 Peking University Shenzhen Hospital, Department of Clinical Laboratory, Shenzhen, China

**Keywords:** thrombus markers, respiratory failure, thrombus formation, prognosis, disease severity, markeri tromba, respiratorna insuficijencija, formiranje tromba, prognoza, ozbiljnost bolesti

## Abstract

**Background:**

This work assessed the value of novel thrombus markers-thrombin-antithrombin complex (TAT), plasmin-a2-plasmin inhibitor complex (PIC), thrombomodulin (TM), and tissue plasminogen activator-inhibitor complex (t-PAIC) applied in patients with respiratory failure (RF), including their role in predicting thrombus formation, evaluating prognosis, and assessing disease severity.

**Methods:**

Eighty patients with RF were enrolled and categorized into mild (n = 10), moderate (n = 9), and severe (n = 71) groups based on disease severity. Meanwhile, patients were also classified into thrombus (n = 14) and non-thrombus (n = 76) groups based on the presence of thrombus. Furthermore, they were assigned into survival (n = 70) and death (n = 20) groups based on prognosis. Traditional coagulation indicators, thrombus markers, infection-related parameters, and respiratory-related indicators were compared among patients in different groups. This work explored the predictive effects of these indicators on the degree of respiratory failure, thrombus formation, and prognosis in various patient groups. Additionally, correlations of thrombus markers and traditional coagulation indicators to respiratory-related indicators and infectionrelated indicators were analyzed.

**Results:**

Upon admission, levels of thrombin-antithrombin complex (TAT), plasmin-a2-plasmin inhibitor complex (PIC), and tissue plasminogen activator-inhibitor complex (t-PAIC) in the thrombus group were sharply higher in contrast to those in the non-thrombus group, showing obvious differences (P<0.05). Patients in the death group experienced remarkably elevated TAT, PIC, t-PAIC, thrombomodulin (TM), and to the survival group (P<0.05). In addition, high-sensitivity C-reactive protein (hs-CRP) in the death group was higher to that in the survival group (P<0.05). Platelet count (PLT) and procalcitonin (PCT) were sharply lower in the survival group (P<0.05). In groups of varying severity, PCT exhibited an elevated level in the severe, demonstrating great differences to the mild to moderate groups (P<0.05). Besides, TAT, PIC, TM, and t-PAIC showed higher sensitivity and accuracy in predicting severe RF, with higher specificity in predicting thrombus formation in RF patients. In correlation analysis, a positive correlation was observed between TT, PCT, and the fraction of inspired oxygen (FiO2). The activated partial thromboplastin time (APTT), PCT, and FiO2 exhibited positive correlations. Additionally, a positive association existed between fibrinogen (FIB), hs-CRP, and PLT. A positive link was identified between D-dimer and hs-CRP, PIC and PLT, as well as tPAIC and PCT.

**Conclusions:**

Thrombus markers exerted a crucial effect in patients experiencing respiratory failure, serving as pivotal indicators for assessing the severity of the condition, identifying thrombotic risk, and predicting prognosis.

## Abstract (sr)

**Uvod:**

U ovom radu je procenjena vrednost novih markera tromba - trombin-antitrombin kompleks (TAT), plazmina2-plazmin inhibitorski kompleks (PIC), trombomodulin (TM) i tkivni plazminogen aktivator-inhibitor kompleks (t-PAIC) primenjenih u pacijenti sa respiratornom insuficijencijom (RF), uključujući njihovu ulogu u predviđanju formiranja tromba, proceni prognoze i proceni težine bolesti.

**Metode:**

Osamdeset pacijenata sa RF je uključeno i kategorisano u blage (n = 10), umerene (n = 9) i teške (n = 71) grupe na osnovu težine bolesti. U međuvremenu, pacijenti su takođe klasifikovani u grupe sa trombom (n = 14) i bez tromba (n = 76) na osnovu prisustva tromba. Staviše, oni su raspoređeni u grupe preživljavanja (n = 70) i smrti (n = 20) na osnovu prognoze. Upoređeni su tradicionalni indikatori koagulacije, markeri tromba, parametri vezani za infekciju i respiratorni indikatori među pacijentima u različitim grupama. Ovaj rad je istraživao prediktivne efekte ovih indikatora na stepen respiratorne insuficijencije, formiranje tromba i prognozu kod različitih grupa pacijenata. Pored toga, analizirane su korelacije markera tromba i tradicionalnih indikatora koagulacije sa respiratornim indikatorima i indikatorima vezanim za infekciju.

**Rezultati:**

Po prijemu, nivoi trombin-antitrombin kompleksa (TAT), plazmin-a2-plazmin inhibitornog kompleksa (PIC) i tkivnog aktivator-inhibitorskog kompleksa plazminogena (t-PAIC) u grupi sa trombom bili su naglo viši u odnosu na one u grupa bez tromba, pokazujući očigledne razlike (P<0,05). Pacijenti u grupi smrti imali su značajno povišene TAT, PIC, t-PAIC, trombomodulin (TM) IU grupi za preživljavanje (P<0,05). Pored toga, visokoosetljivi C-reaktivni protein (hs-CRP) u grupi umrlih je bio veći nego u grupi za preživljavanje (P<0,05). Broj trombocita (PLT) i prokalcitonin (PCT) bili su značajno niži u grupi za preživljavanje (P<0,05). U grupama različite težine, PCT je pokazao povišen nivo u teškim, pokazujuči velike razlike u odnosu na blage do umerene grupe (P<0,05). Osim toga, TAT, PIC, TM i t-PAIC su pokazali veču osetljivost i tačnost u predviđanju teške RF, sa večom specifičnošču u predvi - đanju formiranja tromba kod pacijenata sa RF U korelacionoj analizi uočena je pozitivna korelacija između TT, PCT i frakcije udahnutog kiseonika (FiO^). Aktivirano parcijalno tromboplastinsko vreme (APTT), PCT i FiO^ su pokazali pozitivne korelacije. Pored toga, postojala je pozitivna povezanost između fibrinogena (FIB), hs-CRP i PLT. Identifikovana je pozitivna veza između D-dimera i hs-CRP PIC i PLT, kao i t-PAIC i PCT.

**Zaključak:**

Markeri tromba su imali ključni efekat kod pacijenata sa respiratornom insuficijencijom, služeči kao ključni indikatori za procenu težine stanja, identifikaciju trombo - tičkog rizika i predviđanje prognoze.

## Introduction

Respiratory failure (RF) is a common critical condition characterized by the loss of the body’s oxygenation and carbon dioxide elimination functions, and its severity can lead to a life-threatening situation. Patients with RF exhibit symptoms such as difficulty breathing, hypoxemia, and hypercapnia [1] [2] [3]. RF can be caused by various factors, including acute respiratory distress syndrome, chronic obstructive pulmonary disease, pulmonary embolism, and more. Due to the pathophysiology of respiratory failure diseases, patients with respiratory failure have a high risk of developing coagulation dysfunction [4] [5]. The occurrence of coagulation complications significantly affects the prognosis of patients. Therefore, the management and intervention of thrombotic risk in respiratory failure are crucial. In the clinical practice of treating patients with respiratory failure, it is observed that patients are in a more pronounced hyper-coagulable state, with vascular endothelial damage. This is closely related to the etiology and severity of the patient’s condition. Currently, there is limited research on coagulation indicators and vascular endothelial damage markers for a large population of patients with respiratory failure. This study aims to focus on observing the changes in traditional coagulation indicators and novel thrombotic markers in patients with respiratory failure. The goal is to further explore the value of new molecular markers in the management of coagulation complications in patients with respiratory failure.

Thrombus formation is a common complication in patients experiencing with RF. Excessive bloodcoagulation and platelet activation in the blood can lead to the formation of intravascular thrombi, thereby triggering serious complications such as pulmonary embolism and deep vein thrombosis [6] [7]. Therefore, timely identification of high-risk patients for thrombus formation and implementation of preventive measures are crucial. Traditional thrombus screening methods include platelet count, coagulation function indicators, and D-dimer measurements, but these indicators have certain limitations. In recent years, there has been increasing focus on the application of thrombus markers in respiratory severe disease [8] [9] [10] [11] [12] [13]. Thrombotic markers refer to molecular indicators associated with the physiological processes of blood coagulation, thrombus formation, and thrombus dissolution. In a broad sense, thrombotic markers include D-dimer (DD), von Willebrand factor (vWF), antithrombin III (AT III), fibrinogen (FIB), and others. Novel thrombotic markers specifically refer to indicators such as thrombin-antithrombin complex (TAT), lasmin-α2-plasmin inhibitor complex (PIC), thrombomodulin (TM), and tissue plasminogen activatorinhibitor complex (t-PAIC). The levels of these novel thrombotic markers primarily reflect key processes in the thrombus formation, including a hypercoagulable state, fibrinolysis activation, and vascular endothelial damage. As a result, these markers become valuable indicators for predicting the risk of thrombus formation and rethrombosis [6] [14] [15] [16]. In patients with RF, the application of thrombus markers holds significant clinical significance. Thrombus markers aid in the early identification of high-risk patients for thrombus formation, allowing for targeted preventive measures. They can also be applied to assist in assessing the severity of the underlying disease and predicting the prognosis. Often, the more severe the underlying disease, the more significant the changes in thrombotic markers in patients. As a novel diagnostic and monitoring tool, thrombus markers have the potential for application in patients with RF.

This study aims to explore the application value of thrombus markers in patients suffering from RF. By analyzing and comparing the variations in levels of different types of thrombus markers in RF patients and contrasting them with traditional thrombus screening indicators, we aimed to investigate the predictive value of thrombotic markers for risk stratification, thrombus occurrence, and prognosis in respiratory failure patients. The results herein were expected to provide new guidance for the management and treatment of RF patients, improving patient prognosis and quality of life. Additionally, it may offer new insights and methods for assessing and treating thrombotic risks in patients, contributing to advancements in the field.

## Materials and methods

### Research objects

From August 1, 2022, to July 31, 2023, a total of 90 cases of respiratory failure patients admitted to our ICU were included in this work. These patients had a duration of admission to the department of ≥ 1 day and had records of the thrombus markers upon admission. The study aimed to investigate the predictive value of thrombotic markers for risk stratification, thrombus occurrence, and prognosis in respiratory failure patients. Patients were grouped based on the oxygenation index (PaO_2_/FiO_2_) to assess the severity of their conditions, with 10, 9, and 71 cases in the mild (PaO_2_/FiO_2_ > 300 mmHg), moderate (200 mmHg ≤ PaO_2_/FiO_2_ ≤ 300 mmHg), and severe (PaO_2_/FiO_2_ < 200 mmHg) groups, respectively. They were also categorized into thrombus and non-thrombus group, with 14 and 76 cases, respectively, based on thrombus formation. In addition, they were assigned into survival group (70 cases, not deceased during hospitalization) and death group (20 cases, deceased during hospitalization) based on prognosis. The average age and male-to-female ratio of patients in the mild group were 74.30 ± 6.85 years and 8:2, respectively; those in the moderate group were 61.00 ± 6.33 years and 6:3, respectively; and those in the severe group were 66.65 ± 6.25 years and 44:27, respectively. Patients in the thrombus group had an average age of 68.86 ± 6.82 years and a male-tofemale ratio of 8:6; while those in the non-thrombus group exhibited an average age of 64.22 ± 6.67 years and a male-to-female ratio of 50:26. The average age and male-to-female ratio of patients in the survival group were 63.54 ± 6.12 years and 41:29, respectively; whereas those in the death group were 69.85 ± 6.37 years and 17:3, respectively. Differences in age and gender among patients in various groups were not great (P>0.05), ensuring comparability.

Patients enrolled herein had to satisfy all the following conditions: (1) Diagnosed with respiratory failure (PaO_2_ <60 mmHg, or accompanied by PaCO_2_ > 50 mmHg); (2) comprehensive medical records were available; (3) patients did not have any other infectious diseases; and (4) patients and their families had signed informed consent forms.

Patients with any of following conditions had to be excluded: (1) those with concomitant significant organ-related diseases; (2) individuals with malignant tumors; (3) patients with genetic disorders; and (4) those unwilling to participate in this study.

### Methods

A cross-sectional study design was employed in this research. Retrospectively, patient informationsuch as age, gender, and relevant medical history, including the first and last thrombotic markers, coagulation indicators, inflammatory markers, and respiratory indicators post-admission, was collected from the hospital information system (HIS) and laboratory information system (LIS). The inclusion and exclusion criteria were used in data collection. The analysis involved evaluating traditional coagulation indicators, thrombus markers, infection-related indicators, and respiratory-related indicators among different groups of patients. The aim was to analyze the accuracy, specificity, and sensitivity of these indicators in predicting the severity of RF, thrombus formation, and prognosis. Furthermore, this work compared the predictive effects of different indicators and their combined use. Correlation analyses were conducted between thrombus markers and traditional coagulation indicators, as well as between respiratory-related and infection-related indicators. This exploration aimed to assess the value of combining new biomarkers with traditional ones in specific ICU clinical practices.

### Observation parameters

(1) The general information of patients in each group, including gender and age, was compared.

(2) The traditional coagulation indicators were comparatively analyzed by collecting data on plasma prothrombin time (PT), activated partial thromboplastin time (APTT), thrombin time (TT), fibrinogen (FIB), and D-dimer from patient medical records.

(3) The thrombus markers were compared by collecting data on thrombin-antithrombin complex (TAT), plasmin-a2-plasmin inhibitor complex (PIC), thrombomodulin (TM), and tissue plasminogen activator-inhibitor complex (t-PAIC) from patient medical records.

(4) The infection-related indicators were measured by collecting data on procalcitonin (PCT), high-sensitivity C-reactive protein (hs-CRP), and platelet count (PLT) from patient medical records

(5) The respiratory-related indicators were determined collecting data on arterial oxygen partial pressure (PaO_2_), fraction of inspired oxygen (FiO_2_), arterial carbon dioxide partial pressure (PaCO_2_), and PaO_2_/FiO_2_ from patient medical records.

(6) This work analyzed the accuracy, specificity, and sensitivity of traditional coagulation indicators,thrombus markers, infection-related indicators, and respiratory-related indicators in predicting the severity of RF, thrombus formation, and prognosis for different patient groups. Sensitivity, specificity, and accuracy were calculated using Equations (1), (2), and (3), respectively, where PB referred to the number of true negatives, PM represented the number of true positives, DB denoted the number of false negatives, and DM signified the number of false positives.


(1) Sensitivity= \frac{DM}{PM} \times 100 \%

(2) Specificity= \frac{DB}{PB} \times 100 \%

(3) Accuracy= \frac{DM+DB}{PB+PM} \times 100 \%

(7) ROC curves for different indicators were generated to assess their predictive performance for the prognosis, disease severity, and thrombus formation in patients with RF. Additionally, the correlations between thrombus markers, traditional coagulation indicators, respiratory-related indicators, and infection-related indicators were analyzed.

### Statistical analysis

Data were recorded and summarized using Excel 2016 and statistically processed with Statistic Package for Social Science (SPSS) 20.0 (IBM, Armonk, NY, USA). Descriptive statistics, represented as mean ± standard deviation, were used for continuous variables, which were compared employing ttests. Percentages were used for categorical data, which were analyzed using the chi-square (χ^2^) test. Pearson correlation analysis was employed to examine the relationships among various indicators, with statistical significance set at *P*<0.05. To assess the predictive performance of different indicators for the prognosis, disease severity, and thrombus formation in patients with RF, ROC curves were generated.

## Results

### Differences in traditional coagulation indicators and thrombus markers for patients in different groups

**Figure 1:**
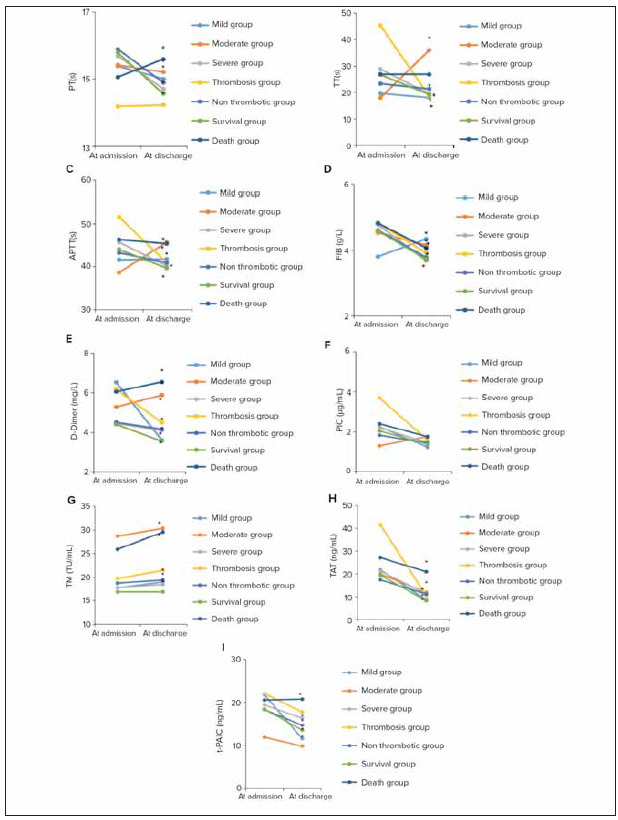
Analysis on traditional coagulation indicators and thrombus markers (A: PT; B: TT; C: APTT; D: FIB; E: D-Dimer; F: PIC; G: TM; H: TAT; I: t-PAIC). (* indicated a significant difference compared to admission, P < 0.05).

Overall, the values of coagulation indicators for patients at the time of discharge showed a significant decrease compared to those during hospitalization. The differences in coagulation indicators between discharge and admission were statistically significant (*P*<0.05). Figure 1 illustrated the comparative analysis of traditional coagulation indicators and thrombus markers among different patient groups. In Figure 1A, the PT of patients in the Death group was significantly higher than that of the Survival group. In Figure 1B, for the Moderate group, the TT at discharge was significantly higher than at admission (*P*<0.05). In Figure 1C, APTT for patients in theModerate group at discharge was significantly higher than at admission (*P*<0.05). In Figure 1D, FIB for patients in the Mile group at discharge was significantly higher than at admission (*P*<0.05). In Figure 1E, the D-Dimer for patients in the Death group was significantly higher than that of the Survival group (*P*<0.05).

As observed, upon admission, patients in the thrombus group exhibited higher PIC, TAT, and t-PAIC in contrast to the non-thrombus group, exhibiting visible differences (*P*<0.05). Additionally, patients in the death group experienced significantly elevated TAT, PIC, TM, and t-PAIC presenting remarkable differences to those in the survival group (*P*<0.05) (Table 1).

**Table 1:** Comparative analysis of clinical indexes in six groups. Note: AA: At admission, AD: At discharge, PT: Prothrombin Time, TT: Thrombin Time,APTT: Activated Partial Thromboplastin Time,FIB: fibrinogen, PIC: Plasmin-a2-plasmin Inhibitor Complex, TM: Thrombomodulin, t-PAIC: Tissue Plasminogen Activatorinhibitor Complex,,TAT: Thrombin-Antithrombin Complex

Index	PT		TT		APTT		FIB		DD		PIC		TM		TAT		t-PAIC	
Group	AA	AD	AA	AD	AA	AD	AA	AD	AA	AD	AA	AD	AA	AD	AA	AD	AA	AD
Mild group	15.38	15.00	19.58	17.95	41.56	41.72	3.81	4.35	6.52	3.62	2.25	1.23	17.71	18.92	21.66	8.48	21.75	11.59
Moderate<br>group	15.41	15.20	17.90	35.94	38.57	45.60	4.54	4.16	5.30	5.89	1.29	1.76	28.68	30.30	19.83	11.69	11.92	9.84
Severe<br>group	15.68	14.70	28.79	19.32	45.63	40.13	4.78	3.68	4.46	4.10	2.21	1.45	17.75	18.41	21.11	11.16	19.39	16.32
Thrombosis<br>group	14.18	14.22	45.31	18.42	51.53	40.86	4.84	3.92	6.18	4.51	3.71	1.58	19.66	21.39	41.51	10.32	22.02	17.76
Non<br>thrombotic<br>group	15.88	14.89	23.24	21.28	43.18	40.85	4.62	3.78	4.52	4.17	1.83	1.43	18.69	19.34	17.27	11.02	18.33	14.67
Survival<br>group	15.78	14.55	26.63	19.15	43.97	39.59	4.60	3.73	4.41	3.56	2.04	1.37	16.83	16.84	19.32	8.08	18.43	13.56
Death<br>group	15.05	15.59	26.82	26.73	46.23	45.29	4.83	4.07	6.05	6.54	2.40	1.75	25.86	29.52	27.07	20.85	20.54	20.73

### Differences in respiratory-related and infectionrelated indicators

**Figure 2:**
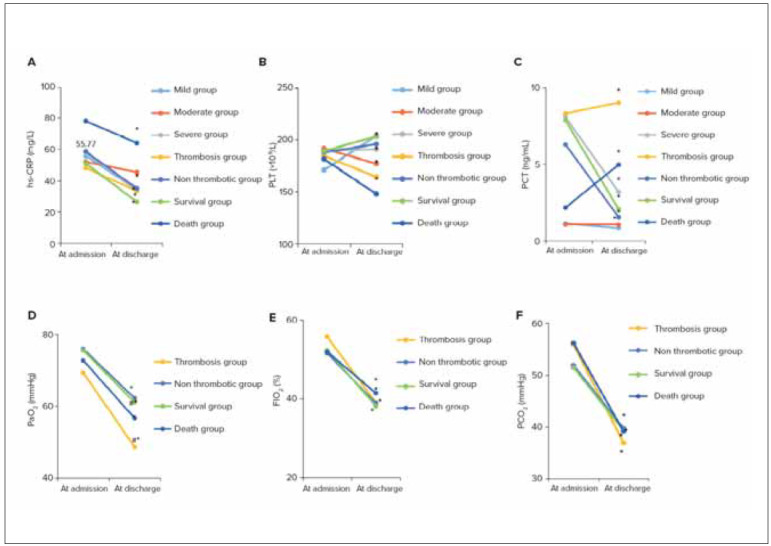
Comparison on respiratory-related and infection-related indicators (A: hs-CRP; B: PLT; C: PCT; D: PaO_2_; E: FIO_2_; F: PCO_2_) (* indicated a significant difference compared to admission, P < 0.05; # indicated a significant difference between thrombus and non-thrombus groups, as well as between death and survival groups, P < 0.05).

Figure 2 depicted the comparative analysis of respiratory-related indicators and infection-related indicators among varying patient groups. It was evident that patients in the severe RF group experienced remarkably higher PCT when comparing to those in the mild to moderate RF group, showing observable differences (*P*<0.05). Additionally, patients in the death group possessed greatly upregulated hs-CRP, demonstrating obvious differences with those in the survival group (*P*<0.05), while PLT and PCT were significantly downregulated in the survival group (*P*<0.05).

In Figure 2D, Figure 2E, and Figure 2F, the differences in PaO_2_, FIO_2_, and PCO_2_ at the time of discharge were statistically significant compared to admission, respectively (*P* < 0.05). There was a significant difference in PaO_2_ between the thrombus group and the non-thrombus group (*P* < 0.05), with the survival group having significantly higher PaO_2_ levels than the death group (*P *< 0.05). There were no significant differences in FIO_2_ and PCO_2_ between the thrombus group and the non-thrombus group (*P* > 0.05).

### Prediction effect of different indicators on moderate and severe RF

**Figure 3:**
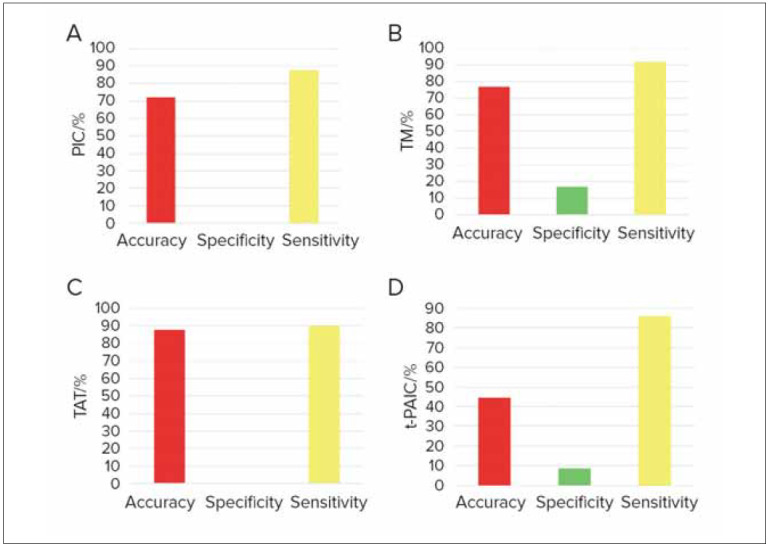
Prediction effect of thrombus markers on moderate and severe RF (A: PIC; B: TM; C: TAT; D: t-PAIC).

Figure 3 presented a comparative analysis of the predictive efficacy of thrombus markers for moderate to severe RF. As depicted, TAT, PIC, TM, and t-PAIC exhibited higher sensitivity in predicting moderate to severe RF. Moreover, TAT, PIC, and TM demonstrated higher accuracy in predicting moderate to severe RF (*P*<0.05). This also suggested a close correlation between the changes in thrombotic marker levels and the worsening of the patient’s condition.

**Figure 4:**
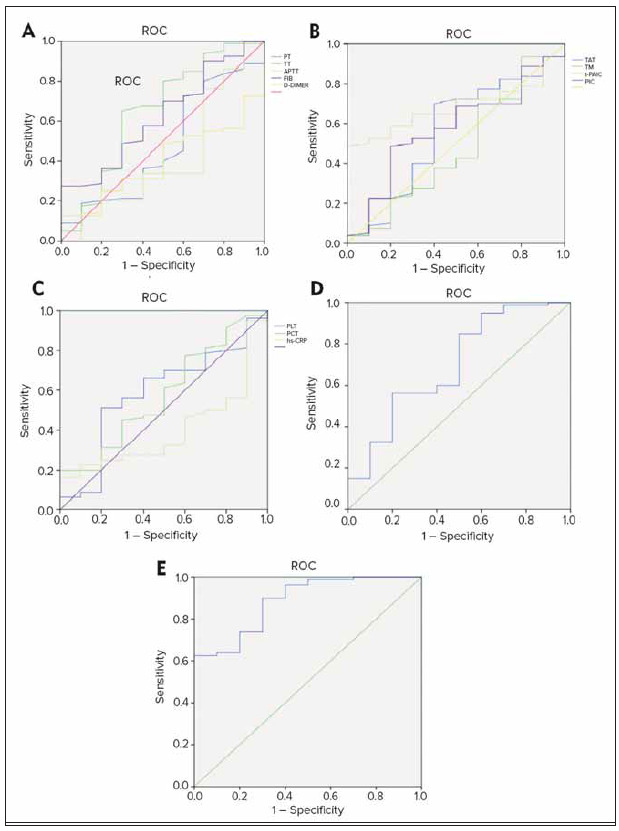
ROC curve for various indicators to predict moderate and severe RF (A: traditional coagulation indicators; B: thrombus markers; C: infection-related indicators; D: traditional coagulation indicators combined with thrombus markers; E: joint prediction of traditional coagulation indicators, thrombus markers, infection-related indicators, and respiratory-related indicators).

Figure 4 illustrated the ROC curve analysis for various indicators in predicting moderate to severe RF. Thrombotic markers exhibited a high diagnostic efficacy in predicting moderate to severe respiratory failure patients. The combination of thrombotic markers with traditional coagulation indicators allowed for a more accurate prediction of the severity of respiratory failure.

### Predictive efficacies of varying indicators on thrombus formation of RF patients

**Figure 5:**
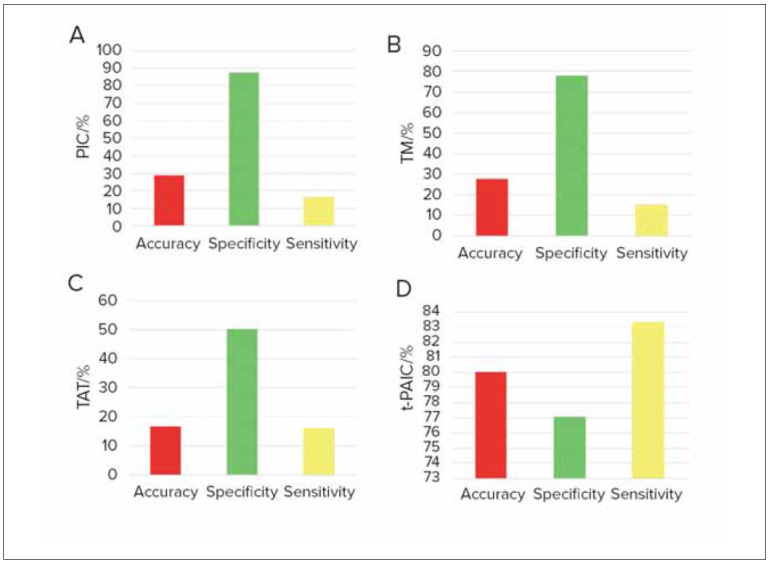
Predictive efficacies of thrombus markers on thrombus formation of RF patients (A: PIC; B: TM; C: TAT; D: t-PAIC).

The predictive efficacies of thrombus markers for thrombus formation in RF patients were compared. Figure 5 signified that TAT, PIC, and TM exhibited high specificity in predicting thrombus formation, while t-PAIC displayed higher sensitivity in it (*P*<0.05).

**Figure 6:**
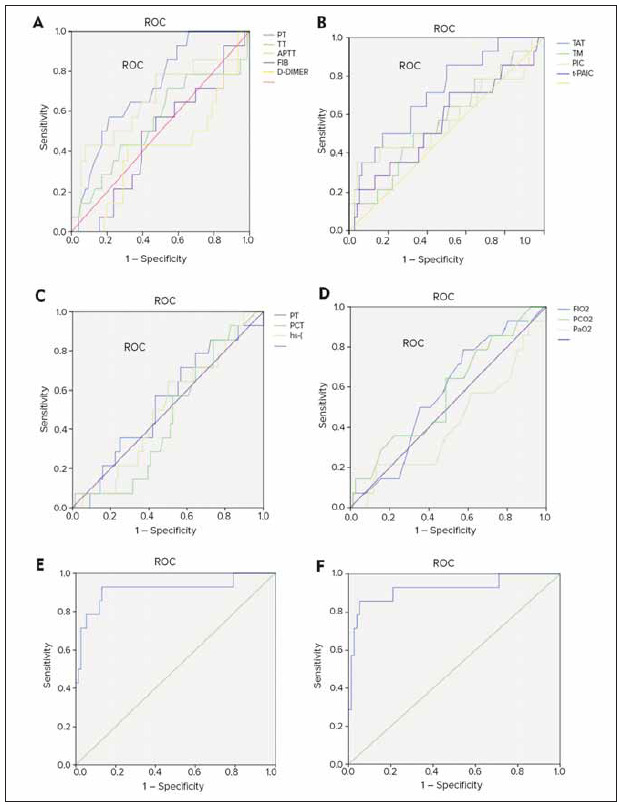
ROC curves for various indicators in predicting thrombus formation in RF patients (A: traditional coagulation indicators; B: thrombus markers; C: infection-related indicators; D: respiratory-related indicators; E: joint prediction of traditional coagulation indicators and thrombus markers; F: combined prediction of traditional coagulation indicators, thrombus markers, infection-related indicators, and respiratory-related indicators).

Figure 6 illustrated the ROC curve analysis for various indicators in predicting thrombus formation in RF patients. As observed, the combined prediction of traditional coagulation indicators and thrombus markers, as well as the use of four types of indicators for combined prediction, has showed effective performance in predicting thrombus formation in RF patients. Therefore, in the management of critically ill patients in the real world, the joint and dynamic monitoring of coagulation markers and thrombus markers can help identify patients’ coagulation abnormalities and thrombotic risks at an earlier stage. This allowed for more precise interventions, reducing the occurrence of coagulation complications.

### Predictive outcomes of various indicators on prognosis of RF patients

**Figure 7:**
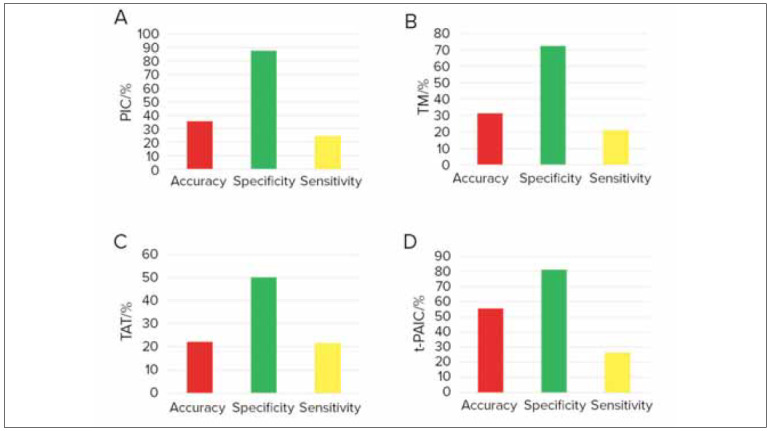
Predictive outcomes of thrombus markers on prognosis of RF patients (A: PIC; B: TM; C: TAT; D: t-PAIC).

The predictive efficacy of thrombus markers for the prognosis of RF patients was compared, as explicated in Figure 7. The results signified that TAT, PIC, TM, and t-PAIC exhibited high specificity in predicting mortality among RF patients (*P*<0.05).

**Figure 8:**
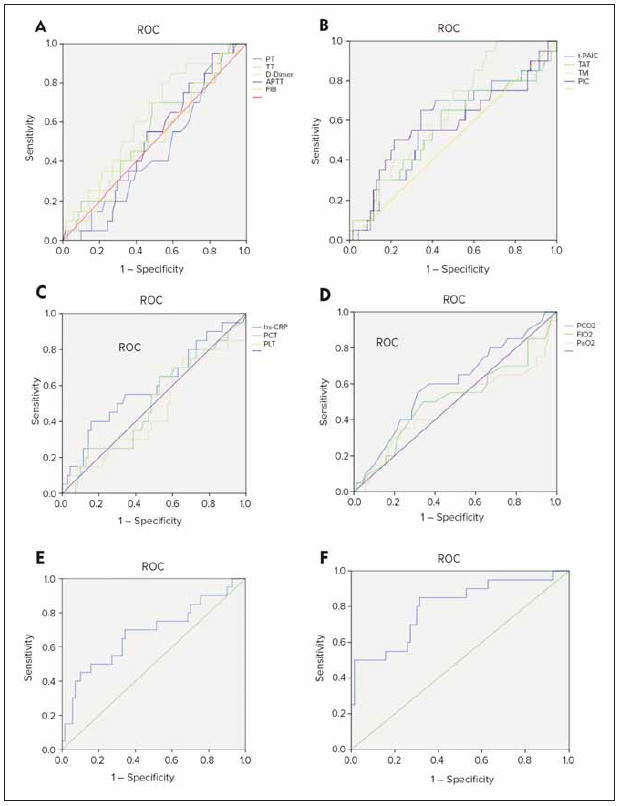
ROC curves for various indicators to predict prognosis of RF patients (A: traditional coagulation indicators; B: thrombus markers; C: infection-related indicators; D: respiratory-related indicators; E: combined predicting results of traditional coagulationindicators and thrombus markers; F: prediction of traditional coagulation indicators, thrombus markers, infection-related indicators, combining with respiratory-related indicators).

Figure 8 illustrated the ROC curve analysis for various indicators in predicting the prognosis of RF patients. In Figure 8A, it can be observed that traditional coagulation indicators fluctuated above and below the standard curve. In Figure 8B, most thrombotic marker indicators were positioned above the standard curve. In Figure 8C, the infection indicator hs-CRP was at the upper end of the standard curve, while other indicators fluctuated above and below the curve. In Figure 8D, respiratory-related indicators initially fluctuated at the upper end of the standard curve and then at the lower end. In Figure 8E, the combined prediction of traditional coagulation indicators and thrombotic markers yielded a ROC curve area greater than that of individual indicators. In Figure 8F, the ROC curve area was maximized. As observed in the figure, the combined prediction using traditional coagulation indicators, thrombus markers, infection-related indicators, and respiratory-related indicators demonstrated effective performance in predicting the prognosis of RF patients. Through the comprehensive analysis of these indicators, it was possible to assess the impact of different factors on the prognosis of RF patients more comprehensively, providing more accurate information for clinical decision-making.

### Correlations of thrombus markers and traditional coagulation indicators to respiratory-related and infection-related indicators

Table 2 represented the correlations between thrombus markers, traditional coagulation indicators, and respiratory-related and infection-related indicators. It was evident that TT and PCT, as well as FIO_2_, exhibited positive correlations. APTT and PCT, along with FIO_2_, also showed positive associations. Additionally, FIB positively correlated with Hs-CRP and PLT. In addition, there existed a positive link between D-Dimer and Hs-CRP, PIC and PLT, as well as t-PAIC and PCT. The results of these correlation analyses strongly suggested that in patients with respiratory failure, the respiratory system, the clotting system, and the immune system interact and influence each other. These findings were also in accordance with the pathophysiology of respiratory failure.

**Table 2:** Correlations of thrombus markers and traditional coagulation indicators to respiratory-related and infection-related indicators. Note: Hs-CRP: High-sensitivity C-reactive Protein, PLT: Platelet Count, PaO_2_: Arterial Oxygen Partial Pressure, FiO_2_: Fraction of Inspired Oxygen, PaCO_2_: Arterial Carbon Dioxide Partial Pressure

		Hs-CRP	PLT	PCT	PaO2	FIO2	PCO2
PT	r	-0.075	-0.1	0.121	-0.025	0.154	0.071
	P	0.481	0.348	0.257	0.817	0.149	0.504
TT	r	0.024	-0.152	0.264	-0.094	0.233	-0.023
	P	0.821	0.153	<0.05	0.379	<0.05	0.827
APTT	r	-0.046	-0.201	0.407	-0.067	0.246	-0.079
	P	0.667	0.058	<0.05	0.528	<0.05	0.462
FIB	r	0.382	0.401	0.134	-0.119	0.179	-0.093
	P	<0.05	<0.05	0.207	0.265	0.091	0.383
D-Dimer	r	0.223	-0.122	-0.024	0.08	-0.092	0.102
	P	<0.05	0.253	0.82	0.452	0.388	0.341
TAT	r	0.022	-0.053	0.04	-0.088	-0.088	0.097
	P	0.838	0.62	0.706	0.411	0.411	0.363
TM	r	0.011	0.06	0.116	0.154	0.058	0.059
	P	0.921	0.575	0.278	0.147	0.585	0.58
PIC	r	-0.06	0.216	-0.168	-0.014	-0.093	0.05
	P	0.573	<0.05	0.114	0.894	0.382	0.638
t-PAIC	r	0.003	-0.171	0.518	0.017	0.061	-0.095
	P	0.974	0.107	<0.05	0.874	0.569	0.372

## Discussion

Thrombus markers provide certain auxiliary guidance for assessing the disease severity in patientswith RF. The findings in this work unveiled that TAT, PIC, TM, and t-PAIC exhibited higher sensitivity in predicting moderate to severe RF, while TAT, PIC, and TM demonstrated elevated accuracy in predicting moderate to severe RF (*P*<0.05). Thrombotic markers provided a certain reference value for assessing the severity of diseases in RF patients but cannot replace the diagnostic function of respiratory-related indicators. These results also strongly suggested that as the underlying disease of the patient becomes more severe, the coagulation-fibrinolysis system of the patient became more abnormal, and their thrombotic markers exhibited more significant abnormalities.

The results of this work indicated that TAT, PIC, and TM exhibit higher specificity in predicting thrombus formation in RF patients, while t-PAIC shows better sensitivity (P<0.05). The results for TAT and PIC aligned with previous findings, but TM and t-PAIC showed differences in their value for assessing thrombotic risk compared to previous reports [17] [18]. This difference can be attributed to the focus of this work on patients with respiratory failure who exhibit significant vascular endothelial damage, resulting in higher levels of TM and t-PAIC. The combined prediction of traditional coagulation indicators and thrombotic markers, as well as the combination of the four categories of indicators, achieved good results in predicting thrombus formation in RF patients. This underscores the significant value of thrombotic markers in predicting thrombus formation. Many clinical studies indicates that the elevated levels of TAT are associated with factors related to thrombus formation, such as pulmonary embolism and deep venous thrombosis [15] [16] [17] [18]. TAT is a complex of thrombin and antithrombin, and an increase in TAT levels reflects the generation of thrombin and activation of the coagulation system [6] [14] [15] [19]. In RF patients, factors such as immune-inflammatory responses and blood stasis may lead to increased coagulation system activity, resulting in thrombus formation [4] [5]. In RF patients, endothelial damage can activate clotting factors, leading to an increase in TAT levels and triggering thrombus formation. The fibrinolysis system dissolves fibrin through plasmin, playing a crucial role in preventing thrombus formation. RF patients often experience inflammatory responses, and the release of inflammatory factors can disrupt the regulation of the fibrinolysis system. There are reports indicating that the fibrinolysis function in RF patients may be inhibited, hindering fibrinolysis reactions and making it difficult for thrombi to dissolve [4] [5]. PIC is a complex of plasmin and its inhibitor, and an increase in PIC levels reflects the generation of plasmin and activation of the fibrinolysis system [6] [14] [19]. Changes in PIC levels are closely related to the occurrence and development of thrombosis [17] [18]. TM is a transmembrane glycoprotein present on the surface of vascular endothelium, exerting anticoagulant and anti-inflammatory effects on the membrane [8] [10] [20]. In this work, soluble thrombomodulin (sTM) was measured, which mainly reflects the degree of vascular endothelial damage. Some studies confirm that TM is significantly elevated in severe infections and can reflect the poor prognosis of septic patients. When the vascular endothelium is damaged, t-PA and PAI-1 are simultaneously released. t-PAIC is a complex formed by the 1:1 binding of t-PA and PAI-1 in the body [19]. t-PAIC is not only a marker of the fibrinolysis system but also a marker of vascular endothelial damage, reflecting endothelial damage and its correlation with organ dysfunction [21].

In addition, this work demonstrated that TAT, PIC, TM, and t-PAIC exhibited higher specificity inpredicting mortality in RF patients (P<0.05). The combined detection of traditional coagulation indicators, thrombotic markers, infection-related indicators, and respiratory-related indicators can assist clinicians in a more comprehensive and accurate assessment of the patient’s condition, guiding treatment decisions, and improving patient prognosis. Different indicators reflect various aspects of pathophysiological changes. Traditional coagulation indicators reflect changes in coagulation function, thrombus markers reflect the balance of thrombus formation and dissolution, infection-related indicators reflect the degree of inflammatory response, and respiratory-related indicators reflect changes in oxygenation and lung function. By considering these different aspects of indicators collectively, a more comprehensive understanding of the patient’s condition and prognosis can be achieved.

Moreover, there may be mutual correlations and influences among different indicators. This workrevealed positive correlations between TT and PCT, as well as FIO_2_; APTT and PCT, as well as FIO_2_; FIB and Hs-CRP, as well as PLT; D-Dimer and Hs-CRP; PIC and PLT; and t-PAIC and PCT. Infection-induced inflammatory responses can activate the coagulation system, leading to changes in coagulation function and an increased risk of thrombus formation. Thrombus formation, in turn, may result in gas exchange disturbances and deterioration of respiratory function. By combining different indicators, these interconnected changes can be better captured, to understand the pathophysiology of the patients and assess the patient’s progress more accurately.

## Conclusion

As specific coagulation markers, thrombosis markers can sensitively reflect hypercoagulability, hyperfibrinolysis and endothelial injury. The detection of thrombotic markers aided in assessing the severity of respiratory failure in patients, identifying thrombotic risk, and predicting prognosis. The changes in thrombotic markers were closely associated with the progression and prognosis of the patient’s condition. This information aided in adjusting treatment plans and making clinical decisions. Therefor, thrombus markers can be used in critically ill patients, to provide crucial information for the early intervention of thrombotic risk and reducing the occurrence of coagulation complications, in order to improve patient outcomes. A limitation of this work lied in the relatively small sample size, so future research could benefit from large-scale, multicenter clinical studies to validate the application effectiveness of thrombus markers in patients with respiratory failure. Additionally, further exploration of their specific value in guiding treatment and improving patient outcomes is warranted.

## Dodatak

### Limitation

Although this study made some valuable findings in exploring the use of thrombotic markers in patients with RF, the sample size of this study was relatively small, which may affect the statistical significance and generalisability of the results. Moreover, this study used a cross-sectional design, a design that does not allow for the determination of causality. Although we observed changes in thrombotic markers in patients with RF, it was not possible to determine whether these changes were a cause or a consequence of disease progression. Future studies should consider a longitudinal design to better understand the role of these markers in disease progression.

### Summary

This article examines the clinical value of novel thrombotic markers (e.g., TAT, PIC, TM, and t-PAIC) in patients with respiratory failure (RF). The results of the study showed that these markers were significantly elevated in patients with RF, suggesting that they may be important in assessing and managing thrombotic risk in patients with RF. However, the study also suffered from limitations such as small sample size and cross-sectional design, and further studies are needed to validate these findings.

### Conflict of interest statement

All the authors declare that they have no conflict of interest in this work.
